# The impact of online upward social comparisons on cyberbullying in the post-epidemic era: a moderated mediating model

**DOI:** 10.3389/fpsyg.2025.1597985

**Published:** 2025-07-23

**Authors:** Guangjie Yuan, Zihan Cheng, Wei Ju

**Affiliations:** ^1^Faculty of Education, Qufu Normal University, Qufu, China; ^2^College of Psychology, Qufu Normal University, Qufu, China

**Keywords:** upward social comparison, cyberbullying, relative deprivation, belief in a just world, mediation model

## Abstract

**Introduction:**

Against the backdrop of the post-pandemic era, this study systematically examines the antecedents and underlying mechanisms of cyberbullying among college students. Building upon the social comparison theory, we particularly investigate how upward social comparison (USC) contributes to cyberbullying through the dual mediating pathways of cognitive and emotional relative deprivation (RD). Importantly, we further explore the moderating role of belief in a just world (BJW) in this psychological process.

**Methods:**

To test our theoretical framework, we collected survey data from 526 undergraduates and employed structural equation modeling with Bootstrap resampling.

**Results:**

The analysis yielded two major findings: First, upward social comparison (USC) was significantly positively associated with cyberbullying, mediated by cognitive and emotional relative deprivation. Second, belief in a just world (BJW) negatively moderated the relationship between relative deprivation (RD) and cyberbullying. Specifically, both general and personal BJW weakened the positive link between cognitive relative deprivation (CRD) and cyberbullying. Similarly, emotional relative deprivation (ERD) effects were moderated by general and personal BJW.

**Discussion:**

The findings not only expand the applicability of the social comparison theory and BJW but also provide empirical evidence for college mental health education.

## 1 Introduction

During the viral venues COVID-19 pandemic, China implemented measures such as closing public venues and canceling mass gatherings. While effectively controlling viral spread, these measures also shifted people’s daily activities from offline to online environments. According to the 51st Statistical Report on Internet Development in China released by the China Internet Network Information Center (CNNIC), the number of internet users in China reached 1.067 billion by the end of 2022. Compared to the pre-pandemic period (2019), this represents an increase of 213 million users, with internet penetration rising by 14 percentage points (Chen et al., 2024). The significant expansion of internet access, coupled with online learning becoming the primary mode of education during the pandemic, has not only increased college students internet exposure but also exacerbated problematic online behaviors such as cyberbullying (Kee et al., 2022; Sorrentino et al., 2023).

Cyberbullying is defined as intentional, repeated harm, humiliation, threats, or harassment perpetrated through digital devices and media in online environments (Wen et al., 2022). This behavior is characterized by its rapid dissemination and wide reach, where negative content propagate exponentially across platforms, amplifying reputational damage to victims (Aboujaoude et al., 2015; Berne et al., 2019; Sticca and Perren, 2012; Wen et al., 2022). The anonymity afforded of virtual identities further compounds harm by enabling perpetrators to evade accountability, intensifying victims’ psychological distress and helplessness due to difficulties in identifying attackers or seeking redress (Wang et al., 2019). Additionally, the persistence of digital records allows harassment to continue indefinitely, subjecting victims to prolonged or lifelong psychological trauma (Berne et al., 2019; Morea and Calvete, 2022). These attributes distinguish cyberbullying from traditional bullying, as its impacts extend both geographically and temporally (Berne et al., 2019; Sticca and Perren, 2012; Wen et al., 2022). Against this backdrop, this study focuses on exploring the antecedents of cyberbullying and its underlying mechanisms, which could address gaps in the literature on post-pandemic cyberbullying and deepen the academically understanding of this phenomenon.

Festinger’s social comparison theory posits that when individuals lack objective criteria for evaluation, they tend to assess their abilities and values by comparing themselves with others (Festinger, 1954). In online environments, curated self-presentations (e.g., idealized social media posts) increase exposure to USC, making it an inevitable psychological process (Li, 2019; Verduyn et al., 2020). RD, a core construct in social comparison research, occurs when individuals perceive discrepancies in resources, opportunities, or social status relative to others (Kim et al., 2017; Lankford and Silva, 2024). This perceived discrepancies triggers cognitive appraisals (e.g., “I deserve better”) and emotional responses (e.g., envy, resentment), which disrupt psychological equilibrium (Shanshan, 2019; Li, 2019; Verduyn et al., 2020). To compensate, individuals may engage in cyberbullying—a maladaptive coping strategy to restore perceived fairness or alleviate negative affect (Buunk et al., 2003; Callan et al., 2015; Park and Park, 2024; Xu and Li, 2024). Moreover, the BJW, which serves as an individual’s internal cognitive schema regarding the fairness of the world, may play a regulatory role in this process (Pan and Zhao, 2024; Wang et al., 2023; Xiong et al., 2022). This is because the BJW affects the way individuals cope with RD, which may further influence the occurrence of cyberbullying (Pan and Zhao, 2024; Wang et al., 2023; Xiong et al., 2022).

**Figure 1:**
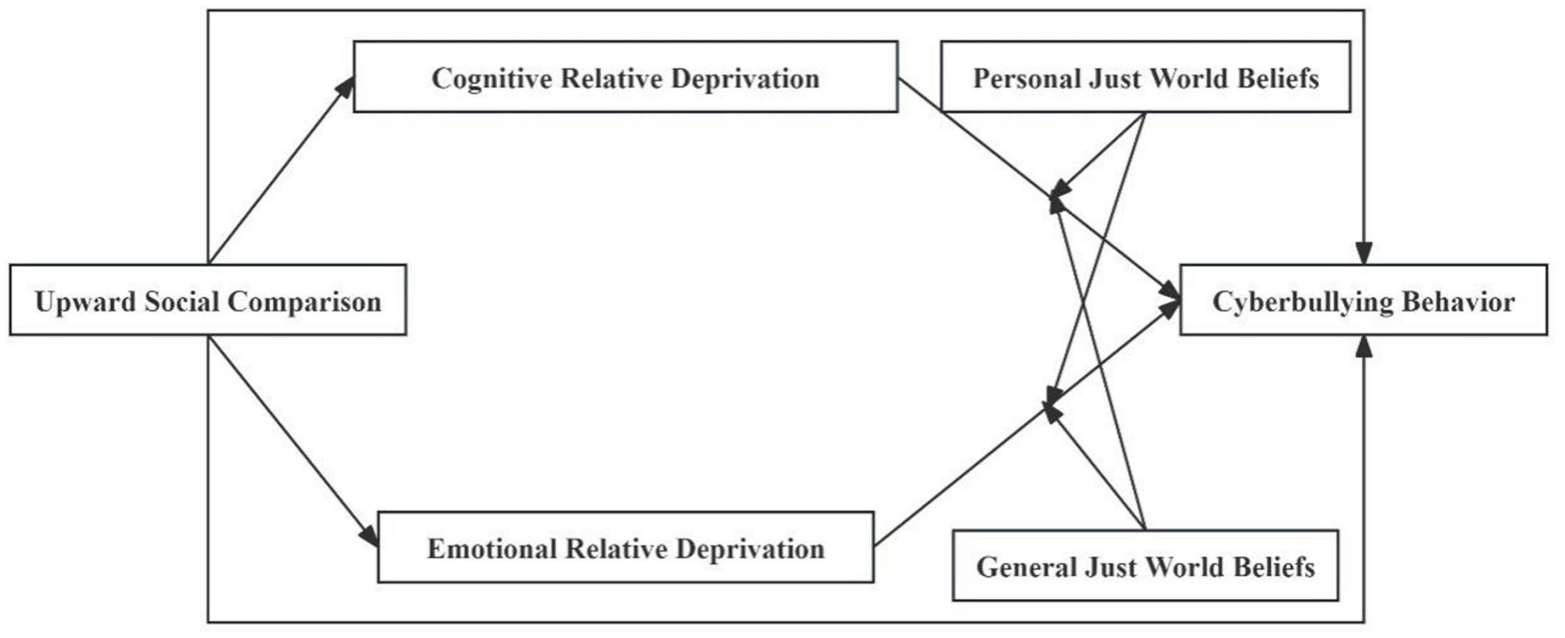
The model of the current study.

Guided by social comparison theory and the belief in a just world (BJW) framework, this study hypothesizes a moderated mediation model (Figure 1) with two core components: (1) USC indirectly influences cyberbullying through dual mediating pathways of CRD (perceived unfair resource allocation) and ERD (negative affect from social discrepancies); (2) BJW moderates the indirect effects of RD on cyberbullying, such that higher BJW attenuates the strength of both cognitive and emotional deprivation pathways.

### 1.1 The impact of USC on cyberbullying

Festinger (1954) social comparison theory posits that individuals have an inherent drive to evaluate their own opinions and abilities, often achieving this through comparisons with others (Festinger, 1954). Social comparisons can be classified into upward, downward, and parallel types based on the relative position of the comparison target (Festinger, 1954; Kong et al., 2021; Taylor and Lobel, 1989). Among these, USC specifically refers to the process where individuals select others who are superior in specific domains (e.g., achievements, resources, or social status) as reference points for self-evaluation (Kong et al., 2021; Li, 2019; Verduyn et al., 2020). With the proliferation of the internet and social platforms, individuals are increasingly exposed to carefully curated information about others’ advantages (e.g., appearance, wealth, career success), leading to more frequent engagement in online USCs.

Online USC is defined as the cognitive process, whether active or passive, wherein individuals assess themselves in relation to digitally-perceived superiors across various dimensions, including but not limited to physical appearance, economic status, and social prestige (Li, 2019; Verduyn et al., 2020). This prevalence stems from two factors: (1) selective information presentation (e.g., social media’s “filtered curation effect”) exaggerates others’ strengths; (2) self-evaluation needs are persistently activated by online social cues (Festinger, 1954; Kong et al., 2021; Li, 2019; Verduyn et al., 2020). Prolonged or excessive online USCs may result in negative psychological outcomes, including reduced self-worth, anxiety/depression (Dou et al., 2023; Lim and Yang, 2015), and even aggressive behavioral tendencies (Barsky, 2011; Livazović and Ham, 2019). For instance, Wen et al. (2022) found a significant positive association between online USC tendencies and cyberbullying, with moral justification mediating this relationship, and the mediating effect being moderated by perceived online social support (Wen et al., 2022).

### 1.2 The mediating role of RD

The concept of RD was originally introduced by American sociologist S. A. Stouffer in his 1949 publication The American Soldier (Davis, 1959; Smith et al., 2012). Subsequent theoretical refinements by R. K. Merton established it as a foundational construct in social psychology and sociology (Bernburg et al., 2009; Davis, 1959). At its core, RD denotes the subjective psychological experience of lacking resources or opportunities when compared to a reference group or standard (Bernburg et al., 2009; Smith et al., 2012). This construct comprises two dimensions: CRD and ERD (Folger, 2014; Smith et al., 2012). CRD, the core component, refers to individuals’ rational assessments of resource/opportunity deficits through social comparisons (Walker and Smith, 2002). ERD, by contrast, represents the emotional consequences of deprivation, such as anger and frustration arising from unmet emotional needs relative to others (Walker and Smith, 2002).

Social comparison functions as the core psychological process underlying RD (Kim et al., 2017; Zhang et al., 2011). Social comparison theory posits that upward comparisons with more advantaged others systematically amplify deprivation by eroding self-evaluations (Kim et al., 2017). The frustration-aggression hypothesis further suggests that individuals often engage in aggressive behaviors to mitigate psychological distress caused by deprivation, seeking to restore emotional equilibrium or enhance self-worth (Berkowitz, 1989; Geen, 1968; Lankford and Silva, 2024). In the digital environment, continuous exposure to curated information exacerbates the frequency of USC. These comparisons may evoke cognitive deprivation (e.g., perceived gaps in resources/achievements) or emotional deprivation (e.g., perceived shortcomings in social relationships). When coupled with the anonymity and reduced accountability of online platforms, these negative emotions arising from the comparisons may escalate into cyberbullying as a coping mechanism. Guided by these theoretical frameworks, we hypothesize:

*H1*: Online USC positively predicts cyberbullying through CRD. Specifically, greater upward comparison will increase cognitive deprivation, which in turn increases the likelihood of cyberbullying.

*H2*: Online USC positively predicts cyberbullying through ERD. Specifically, greater upward comparison will increase emotional deprivation, which in turn increases the likelihood of cyberbullying.

### 1.3 The moderating role of BJW

First proposed by Lerner (1965), BJW posits that individuals fundamentally believe outcomes align with actions (“people get what they deserve”) (Gangloff et al., 2014; Lerner and Miller, 1978). As a core cognitive schema, BJW serves as an interpretive framework for interpreting events and comprises two dimensions: general BJW and personal BJW (Alves and Correia, 2010; Dalbert, 1999). General BJW reflects individuals’ perceptions of fairness at the societal level (e.g., “The world is just overall”), while personal BJW focuses on perceived fairness in one’s personal life (e.g., “I usually get what I deserve”) (Alves and Correia, 2010; Dalbert, 1999). General BJW provides a macro-level framework for understanding social phenomena, whereas personal BJW influences micro-level emotional responses and behavioral decisions in specific contexts (Hafer and Begue, 2005).

BJW plays a critical moderating role in aggressive behavior (Guo and Xia, 2023a; Hafer and Begue, 2005). Individuals with high general BJW believe the world is inherently fair (Lerner and Miller, 1978). When they experiencing CRD, they attribute the resource or opportunity gaps to temporary factors (e.g., “I need to try harder”) rather than systemic injustice. Regarding ERD, individuals with high general BJW rationalize others’ emotional fulfillment (“There’s a reason for their happiness”) and regulate emotions effectively, reducing cyberbullying driven by emotional deficits (Guo and Xia, 2023; Wen et al., 2022). Conversely, individuals with low general BJW blame deprivation on unfairness, using cyberbullying to vent perceived injustice. For personal BJW, high-belief individuals self-reflect and improve when deprived, minimizing cyberbullying; low-belief individuals spiral into negativity, increasing aggression (Guo and Xia, 2023; Wen et al., 2022). Based on this, we hypothesize:

*H3*: General BJW negatively moderates the relationship between CRD and cyberbullying.

*H4*: General BJW negatively moderates the relationship between ERD and cyberbullying.

*H5*: Personal BJW negatively moderates the relationship between CRD and cyberbullying.

*H6*: Personal BJW negatively moderates the relationship between ERD and cyberbullying.

In summary, this study aims to construct a moderated mediation model to systematically explore the mechanism through which online USC influences cyberbullying among college students (Figure 1). Although existing literature has documented the USC-cyberbullying association, two significant research gaps remain unaddressed: (1) the paucity of research examining the differential moderating effects of distinct BJW dimensions (personal versus general) on this relationship, and (2) the insufficient exploration of ERD as a potential mediating mechanism. By systematically investigating these variable relationships, this research seeks to provide a solid theoretical foundation and reliable empirical evidence for preventive intervention efforts targeting cyberbullying among college students. The findings are expected to facilitate the development of more targeted and effective strategies to reduce cyberbullying incidents, ultimately fostering a healthy and harmonious online environment for this population.

## 2 Materials and methods

### 2.1 Participants

This study recruited undergraduates from regular institutions of higher education as participants, utilizing a mixed online and offline approach for data collection. All survey items were completed by self-report. To mitigate common method bias, anonymous questionnaires were administered through three channels: (1) Distribution via social platforms (QQ, WeChat) to acquaintances, resulting in 203 responses, predominantly from southwestern and northeastern China; (2) Offline recruitment of volunteers in university classroom, with 68 responses collected via QR code scanning in classrooms; and (3) Nationwide sampling was conducted via Wen Juan Xing (a leading Chinese online survey platform comparable to Qualtrics in functionality),^1^ yielding 296 valid responses. A total of 567 questionnaires were administered. After excluding invalid responses (e.g., item missingness, high response consistency, regular response patterns, excessively short completion time, inconsistent responses to lie scales), 526 valid questionnaires remained, resulting in an effective response rate of 92.74%. The valid participants ranged in age from 18 to 25 years, with 30.8% being male and 69.2% being female. Grade distribution was as follows: 11.4% freshmen, 15.0% sophomores, 26.8% juniors, and 46.8% seniors. Participants with only-child status accounted for 33.1%, and non-only-child status 66.9%.

### 2.2 Measures

#### 2.2.1 Upward social comparison

The upward comparison subscale compiled by Gibbons and Buunk (1999) and revised by Bai et al. (2013) was adopted (Gibbons and Buunk, 1999; Pan et al., 2023; Bai et al., 2013). This 6-item Likert scale ranged from 1 (strongly disagree) to 5 (strongly agree) includes items such as *“In daily life, I often compare myself with people who are better off than me on social networks”* (original Chinese phrasing retained for cultural fidelity). By explicitly framing items within social networking scenarios, the scale aligns with the study’s focus on online comparison processes. In the current sample, Cronbach’s *α* = 0.907 demonstrated excellent internal consistency, supporting its use in post-pandemic digital contexts.

#### 2.2.2 Cyberbullying behavior

We employed the Cyberbullying Scale developed by Xiuli (2015), which encompasses both direct and indirect forms of cyberbullying. The scale uses a 5-point Likert rating system, where higher scores indicate a greater frequency of cyberbullying. It consists of 12 items, such as “I post aggressive comments on forums and microblogs.” In the present study, the Cronbach’s α coefficient of this scale was 0.934, suggesting excellent internal consistency.

#### 2.2.3 Relative deprivation

The Questionnaire on the RD among College Students, developed by Shanshan (2019); Li (2019), was utilized. This questionnaire comprises two dimensions: cognitive deprivation and emotional deprivation, with a total of 10 items (Li, 2019). Items 2, 4, 6, 8, and 10 were reverse-scored to more comprehensively capture the participants’ genuine feelings (Li, 2019). A 7-point Likert scale is used, and higher scores represent a stronger sense of RD. For example, “Compared with your classmates around you, how do you think you get along with your friends?” In this study, the Cronbach’s α coefficient for the overall scale was 0.928. Specifically, the α coefficient for the CRD sub-scale was 0.893, and for the ERD sub-scale, it was 0.862.

#### 2.2.4 Belief in a just world

We selected the Chinese version of Dalbert’s “BJW Scale,” translated and revised by Su et al. (2012). The scale consists of two dimensions: self-justice and others’ justice, with 7 and 6 items, respectively. It uses a 6-point Likert scale, and there are no reverse-scored items. An example item is “Basically, I think what happens to me is consistent with who I am.” In the current study, the Cronbach’s α coefficient of the entire scale was 0.952. The α coefficient for the personal BJW sub-scale was 0.913, and for the general BJW sub-scale, it was 0.906.

### 2.3 Procedure

This study focused on undergraduate students from universities and used the questionnaire survey method to collect data. Prior to the study, participants were provided with detailed information regarding the research purpose, procedures, measurement tools, potential risks, and benefits. Additionally, a solemn commitment was made to safeguard participants’ personal information. Participants were required to sign an informed consent form and were informed of their right to withdraw from the study at any time during the process. Following this, participants completed demographic information (e.g., gender, age) and subsequently filled out the questionnaire. Upon completion of the study, sincere gratitude was expressed to all participants, who were also compensated appropriately.

## 3 Results

### 3.1 Common method bias test

In this study, Harman’s single-factor test was employed to examine the common method bias. The results indicated that a total of seven factors with eigenvalues >1 were extracted. The first factor accounted for 28.59% of the total variance, which was below the 40% threshold. This indicates the absence of significant common method bias among the variables in this study, and the subsequent data analysis results are highly reliable. This ensures the accuracy and validity for the accuracy and validity of the subsequent research, enabling in-depth analysis and discussion based on these data and more precisely uncover the relationships among USC, RD, BJW, and cyberbullying.

### 3.2 Results of the confirmatory factor analysis

We used AMOS 24 software to conduct a confirmatory factor analysis (CFA) to assess the discriminant validity among the four latent variables: online USC, cyberbullying, RD, and BJW. The corresponding goodness-of-fit indices were also compared. As shown in Table 1, among various factor models, the six-factor model exhibited the best fit (*χ*^2^/df = 1.505, RMSEA = 0.031, CFI = 0.972, TLI = 0.970, SRMR = 0.033). This not only indicates that the sample model fits the data well and can accurately reflect the underlying structure of the data but also suggests that the discriminant validity between the variables is satisfactory. This provides a solid model foundation for the subsequent mediation hypothesis testing, facilitating more accurate testing of the relationships among USC, RD, BJW, and cyberbullying based on the established model.

**Table 1:** Results of the confirmatory factor analysis.

Model	*χ* ^2^	df	*χ*^2^/df	RMSEA	CFI	TLI	SRME
Reference point			<3	<0.080	>0.900	>0.900	<0.080
Single-factor model	2003.083	773	2.630	0.056	0.910	0.904	0.1235
(A + B + C + D + E + F)							
Two-factor model	2033.083	773	2.630	0.056	0.910	0.904	0.1235
(A + B + C + D + E, F)							
Three-factor model	2016.661	772	2.612	0.055	0.911	0.905	0.1229
(A + B + C + D, E, F)							
Four-factor model	1167.934	770	1.517	0.031	0.971	0.970	0.0427
(A + B + C, D, E, F)							
Five-factor model	1167.296	767	1.522	0.032	0.971	0.969	0.0424
(A + B, C, D, E, F)							
Six-factor model	1150.014	764	1.505	0.031	0.972	0.970	0.0329
(A, B, C, D, E, F)							

A, USC; B, CRD; C, ERD; D, PBJW; E, GBJW; F, Cyberbullying.

### 3.3 Results of correlation analysis

The results of the Pearson correlation analysis showed significant positive correlations among USC, cyberbullying, and RD. Specifically, the correlation coefficient between USC and cyberbullying was 0.32 (*p <* 0.001), indicating that the higher the degree of USC among college students, the greater their likelihood of engaging in cyberbullying. The correlation coefficient between USC and RD was 0.38 (*p <* 0.001), suggesting that USC may prompt college students to experience RD. The correlation coefficient between cyberbullying and RD was 0.40 (*p <* 0.001), showing that college students with stronger RD are more inclined to engage in cyberbullying.

Further analysis of the relationships between the cognitive and emotional dimensions of RD and other variables revealed that the correlation coefficient between USC and CRD was 0.31 (*p <* 0.001), and that between USC and ERD was 0.30 (*p <* 0.001). This indicates that USC not only leads college students to perceive they relatively fewer resources or opportunities at the cognitive level but also makes them experience unmet emotional needs at the emotional level, thus leading to RD. These results provide preliminary support for Hypotheses H1 and H3.

Regarding the BJW, its correlations with USC and cyberbullying exhibited different characteristics. BJW was significantly negatively correlated with USC, with a correlation coefficient of −0.19 (*p <* 0.001), indicating that the higher the degree of USC among college students, the lower their level of BJW tends to be. BJW was also significantly negatively correlated with cyberbullying, with a correlation coefficient of −0.34 (*p <* 0.001), suggesting that college students with a higher level of BJW have a lower probability of engaging in cyberbullying. RD was also significantly negatively correlated with BJW, with a correlation coefficient of −0.34 (*p <* 0.001). Specifically, the correlation coefficients between CRD and the general and individual dimensions of BJW were −0.29 (*p <* 0.001) and −0.25 (*p <* 0.001) respectively; the correlation coefficients between ERD and the general and individual dimensions of BJW were −0.31 (*p <* 0.001) and −0.37 (*p <* 0.001) respectively. This shows that the stronger the RD of college students, the lower their level of BJW, and both cognitive and ERD are significantly negatively correlated with the two dimensions of BJW. These results provide preliminary evidence for the verification of Hypotheses H3, H4, H5, and H6.

Through the descriptive statistics and correlation analysis of each variable, this study has preliminarily revealed the relationships among college students’ USC, RD, BJW, and cyberbullying, establishing a foundation for further in-depth exploration of the mechanism of interaction among these variables (Table 2).

**Table 2:** Descriptive statistics of each variable and correlation coefficient matrix.

	M ± SD	Sex	Age	Only Child	Grade	SCP	USC	BJW	CB	RD	CRD	ERD	GBJW	PBJW
Sex	1.69 ± 0.46	1												
Age	21.47 ± 2.7	−0.08	1											
Only Child	1.67 ± 0.47	−0.02	−0.15^***^	1										
Grade	3.09 ± 1.03	0.114^**^	0.32^***^	−0.18^**^	1									
SCP	2.42 ± 0.96	−0.11^*^	−0.04	−0.06	−0.08	1								
USC	20.27 ± 5.06	−0.05	−0.06	0.02	−0.06	0.05	1							
BJW	52.38 ± 12.2	0.01	0.06	−0.02	0.05	0.02	−0.19^***^	1						
CB	19 ± 6.54	−0.06	−0.02	0.05	−0.05	−0.06	0.32^***^	−0.34^***^	1					
RD	34.68 ± 7.98	−0.04	−0.04	0.02	−0.07	−0.03	0.38^***^	−0.34^***^	0.4^***^	1				
CRD	17.46 ± 4.49	−0.053	−0.03	0.03	−0.07	−0.05	0.31^***^	−0.29^***^	0.36^***^		1			
ERD	17.79 ± 4.7	−0.083	−0.03	0.04	−0.09^*^	−0.05	0.3^***^	−0.25^***^	0.31^***^			1		
GBJW	23.3 ± 6.14	0.032	0.07	−0.05	0.08	0.04	−0.13^**^		−0.31^***^	−0.32^***^	−0.35^***^	−0.37^***^	1	
PBJW	28.5 ± 6.6	0.04	0.04	−0.02	0.05	0.04	−0.19^***^		−0.32^***^	−0.37^***^	−0.37^***^	−0.39^***^		1

SCP, Social Class Perception; CB, cyberbullying; M, mean; SD, standard deviation; gender is a dummy variable, female = 2, male = 1; *p < 0.05, **p < 0.01, ***p < 0.001; all values are reserved for two decimal places; the same below.

### 3.4 Results of the mediating effect analysis

In this study, to thoroughly explore the mediating role of CRD, Model 4 was employed Model 4 (Simple Mediating Effect Model) in the PROCESS macro for SPSS and combined it with the bias-corrected percentile bootstrap method for a detailed analysis.

As presented in Table 3, the mediating effect of RD between USC and cyberbullying among college students was significant. Specifically, the value of the mediating effect was 0.251, and the value of the total effect was 0.410. The mediating effect accounted for 61.22% of the total effect. Moreover, neither the upper nor the lower limit of the 95% confidence interval of the mediating effect included 0. This result strongly confirmed the reliability and stability of the mediating role of RD between USC and cyberbullying among college students.

**Table 3:** Test of the mediating effect of RD.

Path	Effect	Percentage	Boot SE	Bootstrapping
				95% CI
USC → CB	TC			
	0.41		0.05	0.30, 0.52
USC → CB	DC			
	0.25	61.2%	0.06	0.14, 0.36
USC → RD → CB	IC			
	0.16	38.8%	0.026	0.11, 0.21
USC → CB	DC			
	0.29	71.5%	0.054	0.19, 0.40
USC → CRD → CB	IC			
	0.12	28.5%	0.025	0.07, 0.17
USC → CB	DC			
	0.32	77.3%	0.06	0.21, 0.42
USC → ERD → CB	IC			
	0.09	22.7%	0.02	0.05, 0.15

CB, cyberbullying; TC, total effect; DC, direct effect; IC, indirect effect.

Subsequently, both RD dimensions (cognitive and emotional) were separately used as mediating variables and tested by the percentile Bootstrap method. The results indicated that both dimensions showed significant mediating effects between USC and cyberbullying among college students. When CRD was used as the mediating variable, the direct effect of CRD was 0.293, accounting for 71.5% of the total effect; when ERD was used as the mediating variable, its direct effect was 0.317, accounting for 77.3% of the total effect. Additionally, the 95% confidence intervals of the mediating effects of both excluded zero. These results verified Hypotheses H1 and H2, thereby providing stronger and compelling evidence for the mediating role of RD between USC and cyberbullying among college students.

### 3.5 Results of the moderating effect test

In this study, Model 14 (the latter part of the mediation model is moderated) in the SPSS macro program was employed to examine the moderating effect of the BJW. After controlling for the grade variable, the BJW was incorporated into the regression equation model. The analysis results revealed that the interaction term between RD and the BJW had a significant predictive effect on college students’ cyberbullying (Table 4). Specifically, b = −0.38, t = −10.38, *p <* 0.001, 95% CI [−0.03, −0.02]. These findings demonstrate that the BJW played a moderating role in the latter part of the path where “college students’ cyberbullying is mediated by RD.”

**Table 4:** The moderating effect of BJW on the relationship between CRD and cyberbullying.

	*Y* (overall moderating effect)			Y (M1*W1)			Y (M1*W2)		
	*β*	*t*	95% CI	*β*	*t*	95% CI	*β*	*t*	95% CI
*X*	0.12	3.22^**^	0.06, 0.26	0.17	4.37^***^	0.12, 0.31	0.15	4.00^***^	0.1, 0.29
*M*	0.20	5.04^***^	0.10, 0.23						
*W*	−0.20	−5.43^***^	−0.15, −0.07						
M*W	−0.38	−10.38^***^	−0.03, −0.02						
*M1*				0.17	4.32^***^	0.14, 0.37	0.18	4.52^***^	0.15, 0.38
*W1*				−0.19	−5.06^***^	−0.29, −0.13			
*W2*							−0.20	−5.15^***^	−0.27, −0.12
M1*W1				−0.34	−9.99^***^	−0.06, −0.04			
M1*W2							−0.38	−10.46^***^	−0.06, −0.04
*R^2^*	0.36			0.34			0.35		
*F*	74.62			66.46			69.43		

Variable notations in this study are defined as follows (full terms provided upon first mention, symbols consistent in subsequent figures/tables). X, USC; Y, cyberbullying; M, RD; M₁, CRD; M₂, ERD; W, BJW; W₁, GBJW; W₂, PBJW.

Subsequently, the GBJW was further incorporated into the regression equation model. The predictive effects of the interaction terms between this dimension and CRD, as well as ERD, on college students’ cyberbullying were separately investigated. The results showed that the interaction term between CRD and the general BJW had a significant predictive effect on college students’ cyberbullying (b = −0.34, t = −9.99, p < 0.001, 95% CI [−0.06, −0.04]). Similarly, the interaction term between CRD and the personal BJW also had a significant predictive effect on this behavior (b = −0.38, t = −10.46, *p <* 0.001, 95% CI [−0.06, −0.04]). Collectively, these results support that both the general belief in a just world (GBJW) and the personal belief in a just world (PBJW) played a negative moderating role in the relationship between CRD and cyberbullying, thus verifying Hypotheses H3 and H5.

Finally, both dimensions of the BJW were incorporated into the regression equation model, and their interactions with ERD were analyzed. The results showed that both interaction terms had a significant negative predictive effect on college students’ cyberbullying (Table 5). Specifically, the GBJW (b = −0.38, t = −10.29, *p <* 0.001, 95% CI [−0.07, −0.15]) played a moderating role in the latter part of the path where ERD influenced college students’ cyberbullying. The PBJW, as a moderating variable, also significantly influenced this path (b = −0.39, t = −10.75, *p <* 0.001, 95% CI [−0.06, −0.04]). This result verified Hypotheses 4 and 6.

**Table 5:** The moderating effect of BJW on the relationship between ERD and cyberbullying.

	Y (M2*W1)			Y (M2*W2)		
	*β*	*t*	95% CI	*β*	*t*	95% CI
*X*	0.18	4.59^***^	0.13, 0.32	0.16	4.21^***^	0.11, 0.30
*M2*	0.14	3.45^**^	0.08, 0.30	0.15	3.67^***^	0.10, 0.31
*W1*	−0.20	−5.14^***^	−0.29, −0.13			
*W2*				−0.20	−5.21^***^	−0.28, −0.13
M2*W1	−0.38	−10.29^***^	−0.07, −0.05			
M2*W2				−0.39	−10.75^***^	−0.06, −0.04
*R^2^*	0.33			0.34		
*F*	63.99			66.87		

## 4 Discussion

### 4.1 The relationship between USC and cyberbullying

This study demonstrated that USC has a significant positive predictive effect on cyberbullying. These findings corroborate empirical research conclusions from scholars such as Lei et al. (2023) and Wen et al. (2022). In terms of the theoretical mechanism, this research validates the core assumption of the self-evaluation maintenance theory. Specifically, when individuals are frequently exposed to selectively presented idealized images of others in virtual spaces, such as carefully curated life narratives on social platforms, USC activates their self-assessment system, leading to a temporary decline in self-worth (Tesser, 1988). The “idealized others” images constructed through digital impression management techniques, encompassing dimensions such as achievement display and quality of life, contrast sharply with an individual’s real self, triggering cognitive dissonance and weakening self-efficacy (Li, 2019; Verduyn et al., 2020).

Building on this mechanism, the psychological mechanism of this negative self-evaluation further takes effect through an emotional mediation path. When individuals perceive the gap between themselves and others, it can trigger negative emotions such as jealousy and hostility (Li, 2019; Verduyn et al., 2020). The dynamic model of the frustration-aggression theory posits that psychological frustration resulting from social comparison activates a preparatory state for aggressive behavior (Leander and Chartrand, 2017). It is important to note that the three characteristics of the virtual space-anonymity of identity, diffusion of responsibility, and disinhibition of behavior-provide an ideal environment for the implementation of aggressive behavior (Aboujaoude et al., 2015; Chu et al., 2018; Malik and Dadure, 2024; Wang et al., 2019). In other words, individuals are more likely to engage in cyberbullying to vent their emotions and achieve psychological compensation under conditions of reduced social accountability.

### 4.2 The mediating role of RD

Through testing the mediating effect, this study found that USC on the Internet significantly predicts cyberbullying through the dual-path mechanism of CRD and ERD. The specific mechanisms are as follows:

*CRD path*: in the post-pandemic Web 3.0 era, individuals are exposed to an average of 15.7 (SD = 6.2) pieces of “idealized self-presentation” content strengthened by algorithms on a daily basis, which consistently triggers USC (Chen et al., 2024; Li, 2019; Verduyn et al., 2020). This cognitive comparison can highlight one’s own deficiencies and disadvantages through the two-dimensional deconstruction mechanism of “real self-ideal self.” The result of this comparison will gradually lead individuals to form a negative self-cognition, specifically manifested as a significant decrease in self-efficacy and a significant increase in the level of depression. These negative psychological states, in turn, have many adverse effects on their daily lives (Dou et al., 2023; Li, 2019; Lim and Yang, 2015). From the perspective of cognitive-behavioral theory, in order to restore the consistency between cognition and behavior, individuals may choose to engage in cyber-aggressive behavior to balance their negative self-cognition and relieve psychological stress (Lin et al., 2024; Wang et al., 2023).

*ERD path:* from the perspective of the generation mechanism of the sense of ERD, USC creates an obvious psychological gap in individuals, which in turn triggers a series of negative emotions such as anxiety, tension, and panic. With the continuous accumulation of these negative emotions, individuals will eventually deeply experience the sense of RD (Folger, 2014; Smith et al., 2012; Song et al., 2024). This strong sense of emotional deprivation will become a driving factor for individual behavior, prompting them to exhibit abnormal behaviors such as aggression and over-reaction on the Internet (Lin et al., 2024; Wang et al., 2023). It is particularly noteworthy that due to the unique characteristics of cyberbullying, such as anonymity, openness, and low accountability, it provides a convenient channel for groups with a relatively strong sense of RD to vent their negative emotions such as anger, dissatisfaction, and a sense of unfairness (Aboujaoude et al., 2015; Chu et al., 2018; Malik and Dadure, 2024; Wang et al., 2019).

In conclusion, this study reveals the mechanism by which USC affects cyberbullying through the two RD paths of cognition and emotion. This finding not only deepens the understanding of the psychological roots of cyberbullying but also provides a theoretical basis for targeted interventions. Future research can be further expanded, including but not limited to how to effectively reduce USC in the online environment or how to help individuals better cope with the sense of RD to reduce the frequency of cyberbullying.

### 4.3 The moderating role of BJW

The results of the moderating effect test indicate that “the GBJW significantly negatively moderates the positive relationships of both CRD and ERD with cyberbullying. Research has shown that individuals with a strong GBJW, when perceiving CRD (i.e., subjectively believing there are disparities in resources and opportunities between themselves and others), tend to adopt internal controllable attributions (Dalbert, 2013). This attribution pattern is rooted in their core belief in the “fundamental justice of the world”—they attribute the disparities in social comparison to “insufficient personal effort” (e.g., “I need to work harder”) rather than “injustice in the social system” (e.g., “The society is unfair to me”) (Lerner and Miller, 1978). Longitudinal studies have revealed that this attribution bias can substantially reduce the level of psychological imbalance (Smith et al., 2012) and diminish the intention of aggressive behavior (Zych et al., 2019). These convergent findings collectively support the “cognitive restructuring hypothesis” of the GBJW (Hafer and Begue, 2005). That is, by converting the inequities in social comparison into controllable personal variables, individuals with a strong GBJW prevent the transformation of CRD into hostile cognition and behaviors.

In the moderating pathway of ERD, individuals with a strong GBJW exhibit a significant emotional buffering effect (Hafer and Begue, 2005). The emotional information-processing theory proposed by Pribram (1967) posits that emotions are essentially the result of the conflict between an individual’s cognitive appraisal of environmental information and their existing belief system (Pribram, 1967). When individuals with a strong GBJW encounter situations of ERD, their belief in the “fundamental justice of the world” may trigger a cognitive reframing process of a “just narrative,” transforming the sense of ERD into adaptive emotions (such as self-motivation) rather than hostile emotions (Clore and Huntsinger, 2007; Gross, 1998). This positive cognitive framework contributes to enhancing emotional management skills and alleviating the adverse effects of negative emotions (Garnefski et al., 2001). When negative emotions are effectively regulated, the occurrence of emotion-driven cyberbullying will correspondingly decrease. This process may be associated with the GBJW enhancing psychological resilience, enabling individuals to maintain good emotional regulation abilities when facing emotional setbacks (Southwick et al., 2014).

In addition, this study also reveals that the PBJW exerts a negative moderating effect on the positive predictive relationships of both CRD and ERD with cyberbullying. CRD, as a dimension of rational evaluation (indicating the perception of unfair resource distribution), can trigger instrumental aggression tendencies (such as attempting to restore a sense of fairness through bullying). However, these tendencies are mitigated by the PBJW. This is highly consistent with the “Just World Compensation Model” proposed by Lerner and Lerner (1980). Individuals with a strong PBJW are more likely to maintain cognitive balance by internalizing justice norms rather than resorting to external aggression (Hafer and Begue, 2005; Lerner and Miller, 1978). ERD, as an emotion-driven dimension (characterized by the experience of anger due to perceived unfairness), can lead to impulsive aggressive behaviors. These behaviors are also buffered by the PBJW. This validates the reverse mechanism of the moral disengagement theory: when individuals firmly believe that “the world is just” (a high level of PBJW), moral self-restraint inhibits emotion-driven deviant behaviors (Bandura et al., 1996; Tangney et al., 2018). Notably, this finding is reinforced by Guo’s (2023) longitudinal study confirming the mediating role of hostile attribution bias in the RD-cyberbullying link (Guo and Xia, 2023). ‌Our study further demonstrates that BJW (particularly the personal dimension) exerts a buffering effect within this pathway, suggesting that cognitive restructuring may inhibit the transformation of hostile emotions into cyberbullying.

In summary, this study found that BJW exerts a negative moderating effect on the relationship between RD and cyberbullying by influencing individuals’ cognitive patterns and emotion regulation abilities. This finding not only extends the theoretical frameworks proposed by Dalbert (2013) and Hafer (2005b), but also reveals the dual mechanisms through which BJW operates: at the cognitive level, it promotes controllable attributions for feelings of deprivation, while at the emotional level, it effectively mitigates the resulting anger (Dalbert and Maes, 2002; Hafer and Begue, 2005).

‌Based on the aforementioned mechanisms, this study proposes a dual-process cognitive-affective prevention framework for cyberbullying.‌ This framework operates through two synergistic mechanisms: First, within the ‌cognitive restructuring‌ dimension, ‌guiding individuals to attribute social disparities to controllable factors, such as personal effort levels, helps mitigate their perceived injustice (Donat et al., 2018).‌ Second, within the ‌emotion regulation‌ dimension, employing ‌ structured emotion regulation training can effectively alleviate the aversive emotional arousal induced by RD (Bartholomaeus et al., 2019).‌ ‌This framework not only establishes a theoretical foundation for subsequent research, but also provides novel theoretical grounding and practical implications for the preventive intervention of cyberbullying.

## 5 Conclusion

Building on social cognitive theory, this study examines underlying psychological mechanisms of cyberbullying among university students in the post-pandemic context through establishing a moderated mediation model. Findings demonstrate that USC predicts cyberbullying occurrence through dual mediating pathways of CRD and ERD. The study further identifies BJW’s significant moderating role in weakening RD’s effects. These findings support an innovative cognitive-emotional prevention framework that advances cyberbullying research while providing evidence-based intervention protocols for higher education. The framework contributes to developing healthy digital campus environments.

‌Despite its contributions, this study has limitations. First, self-reported data may introduce response biases. Future studies should adopt multi-method designs. Second, the exclusive focus on collectivist cultures (e.g., East Asia) limits cross-cultural generalizability. Subsequent research should include diverse cultural samples (vs. individualistic cultures) for comparative analysis.

## Data Availability

The original contributions presented in the study are included in the article/supplementary material, further inquiries can be directed to the corresponding authors.

## Footnotes

^1^ www.wjx.cn
